# Multimodal Assessment Shows a Mostly Preserved Cognitive Status in Incidentally Discovered Low Grade Gliomas: A Single Institution Study

**DOI:** 10.3390/cancers12010156

**Published:** 2020-01-08

**Authors:** Ilaria Guarracino, Tamara Ius, Enrico Pegolo, Daniela Cesselli, Miran Skrap, Barbara Tomasino

**Affiliations:** 1 Scientific Institute, IRCCS E. Medea, San Vito al Tagliamento, 33078 Pordenone, Italy; ilaria.guarracino@lanostrafamiglia.it; 2 Unità Operativa di Neurochirurgia, Azienda Sanitaria Universitaria Integrata S. Maria della Misericordia, 33100 Udine, Italy; tamara.ius@asuiud.sanita.fvg.it (T.I.); skrap@asuiud.sanita.fvg.it (M.S.); 3 Institute of Pathology, Santa Maria della Misericordia University Hospital, 33100 Udine, Italy; enrico.pegolo@uniud.it (E.P.); daniela.cesselli@uniud.it (D.C.)

**Keywords:** low-grade glioma, incidental findings, neuropsychology, brain mapping

## Abstract

Incidentally discovered low-grade gliomas (iLGGs) are poorly reported in the literature. Still less is known about iLGG patients’ neuropsychological profile: It is unclear whether iLGG patients are cognitively proficient, thus further confirming the concept of asymptomatic. From our monoinstitutional cohort of 332 patients operated for LGG from 2000 to 2017 we selected those who underwent a neuropsychological testing (*n* = 217, from 2008 to 2017), and identified 24 young (mean age 38.5 ± 1.06) patients with iLGGs (16 of 24, left hemisphere iLGGs, 8 of 24 right hemisphere iLGGs). The maximum lesions overlap occurred in the left inferior frontal gyrus and in the right anterior cingulate/superior medial frontal gyrus. Patients were cognitively preserved except mild to borderline difficulties in a few of them. The analysis of the equivalent scores (a score laying below or equal to the external nonparametric tolerance limit of adjusted scores corresponding to 0, 1, 2 and 3 are intermediate) highlighted the presence of additional borderline performances. Molecular class correlated with a normal function at visual–spatial intelligence (*p* = 0.05) and at spatial short-term memory (*p* = 0.029). Results indicate that at this time of tumor growth, patients’ cognitive abilities are still functional, but are slowly approaching the borderline level.

## 1. Introduction

An incidental low-grade glioma (iLGG) is defined as a glioma found on imaging studies obtained for a reason unrelated to the underlying tumor [1], such as participation as a volunteer for magnetic resonance imaging (MRI) studies, trauma, headache without associated mass effect, or endocrinologic work up [2]. For instance, among the modes of discovery of iLGGs in the general population undergoing brain imaging, [3] reported headaches (26%), screening (21%), trauma (15%), syncope (8.8%), dizziness (8.8%), hearing loss (5.7%), psychosis (2.9%) and others (4%). In fact, iLGGs remain undiscovered until the subject performs a radiological imaging examination for unrelated reason [4]. It is unclear whether iLGGs patients can be defined as asymptomatic from a neuropsychological perspective, and whether iLGGs remain undiscovered also because the patients do not experience any cognitive symptoms. By contrast, epilepsy is the most common onset symptom of symptomatic LGG, with a seizure frequency ranging from 60 to 90% [5,6,7,8,9,10].

Patients with iLGGs represent an extremely rare clinical subgroup of LGGs; the incidence ranging between 0.04 and 0.2% in the general population [11]. The natural history of iLGGs prior to discovery is poorly understood [2,12], and the question of clinical management has become a topic of increasing interest in current literature [1,2,3,13,14,15,16,17,18,19,20,21]. None of the above-mentioned studies presented neuropsychological data about patients with incidental brain tumors. Nonetheless, a significant aspect is a shortage of neuropsychological data related to this type of patient. There is a single evidence in the literature on cognitive deficits in individuals with iLGGs [14], although the analysis of cognitive functions could be the only clinical parameter of investigation in a group of subjects that did not manifest any symptoms as epileptic seizures. Authors of [14] found cognitive deficits in a group of 15 patients with iLGGs, predominantly in executive functioning, working memory and psychomotor attention. Almost half of the group had a real cognitive deficit and almost two out of three had at least poor cognitive functioning. Moreover, a relationship has been found between the neuropsychological deficit and the complaints reported by patients (80%). Therefore, the absence in these patients of seizures or pharmacological therapies that could be responsible for the cognitive deficits indicate that their cognitive status could depend exclusively on the damage caused by the iLGGs growth [14].

Our aim was to determine the presence of subclinical dysfunctions in patients with iLGGs as revealed by their cognitive framework, and to assess their lesion localization in addition to their molecular data. Our aim was therefore to evaluate the cognitive function to understand if these diagnoses can be considered incidental not only for the standard neurosurgical evaluation but also from a neuropsychological perspective and to analyze their lesion localization. To do so, in this retrospective study we review a cohort of patients with LGGs surgically treated in our institution.

## 2. Results

From our monoinstitutional cohort of 332 patients operated for low-grade gliomas from 2000 to 2018, we selected those who underwent a neuropsychological testing (*n* = 217, from 2008 to 2018), and identified 24 patients (11.52%) who met the criteria for iLGGs (see Table 1 for their clinical and demographic data). The reasons for initial MRI by which the brain tumors were incidentally discovered are summarized in Table 2. None of the patients had a history of neurological or psychiatric disease. Molecular analyses revealed that there were 13 diffuse astrocytoma Isocitrate dehydrogenase (IDH) mutant (54.16%), seven oligodendroglioma, IDH mutant and 1p/19q co-deleted (29.16%), and four diffuse astrocytoma, IDH wildtype (16.66%).

A total of 16 out of 24 (67%) patients had a left hemisphere iLGG, while eight out of 24 (33%) patients had a right hemisphere iLGG.

The average age of the group was 38.5 years ± 1.06; the average education was 14.35 ± 1.9. There wasn’t a prevalence of incidental discovery in female or in male (F = 12; M = 12). One patient was pure left-handed (−100), two mixed right-handed (83%, 50%), one patient was correct left-handed, and all the other patients were pure right-handed (100) as assessed by Edinburgh Handedness Inventory [22].

### 2.1. Neuropsychological Data

#### 2.1.1. Below the Normal Range Performances

For the left hemisphere iLGGs patients (see Table 3 and Table 4) we found that P#5 showed a performance below normal range in verbal short-term memory and in naming verbs, P#1 and P#7 showed a performance below normal range in verbal working memory, and P#1 had a performance below normal range in naming verbs, in writing, and in auditory and visual lexical decisions, P#4 had a selective below normal range in visual and auditory lexical decisions, and P#13 had a performance below normal range in writing words and in visual lexical decisions.

For the right hemisphere iLGGs patients (see Table 3 and Table 4) we found that P#6 had a performance below normal range in visual–spatial memory.

#### 2.1.2. Borderline Performances

We used a five-point scale (0–4), termed equivalent scores. The equivalent scores were derived from the reference articles of each task. A score laying below or equal/above the external nonparametric tolerance limit of adjusted scores corresponds to 0 or to 4 respectively; 1, 2 and 3 are intermediate [23]. By analyzing patients’ equivalent scores to identify borderline performance (see Table 3 and Table 4), we found that, for the left hemisphere iLGGs patients, P#6 and P#8 had an equivalent score of 1 selectively in phonological fluency, while P#1 had an equivalent score of 1 in psychomotor velocity, as did P#7 in verbal short-term memory and P#13 in verbal short-term memory, verbal working memory and in psychomotor velocity.

**Figure 1:**
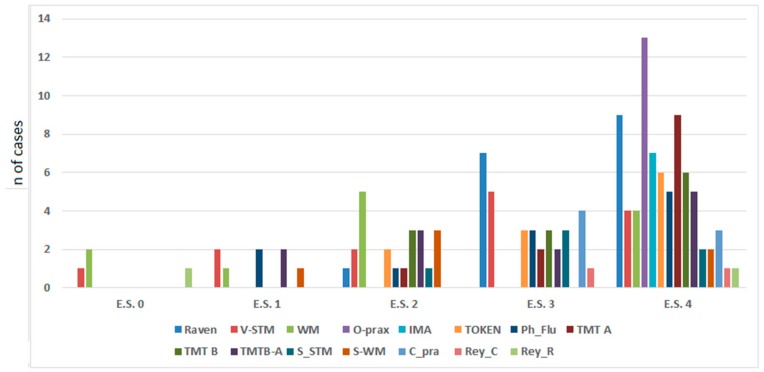
Left hemisphere (LH) and right hemisphere (RH) iLGG patients’ neuropsychological level of performance stratified by their equivalent score, showing a higher distribution of performance in the equivalent scores of 3 and 4 for all the tasks. V_STM = verbal short-term memory; WM = working memory; O_prax = oral praxis; IMA = ideomotor apraxia; Token = Token Test; Ph_Flu = phonological fluency; TMT = Trail Making Test; S_STM = spatial short-term memory; S_WM = spatial working memory; C_pra = constructional apraxia; Rey_C = Rey–Osterrieth complex figure test (Copy immediate); Rey_R = Rey–Osterrieth complex figure test (Remember).

For the right hemisphere iLGGs patients, P#4 had an equivalent score of 1 selectively in visual–spatial short-term memory (see Figure 1).

To sum up, it appears that 18out of 24 (75%) patients were within the normal range performance, three out of 24 (12.5%) had scattered altered performance, and three out of 24 (12.5%) had borderline performance.

#### 2.1.3. Correlation between Patients’ Equivalent Score and Molecular Class

Results show that the patients’ equivalent scores at the Raven test (r(19) = 0.447, *p* = 0.055) and at the visual short-term memory test (r(6) = 0.857, *p* = 0.029) were positively correlated with molecular class, meaning that patients with oligodendroglioma (IDH mutant and 1p/19q co-deleted) had higher equivalent scores than patients with diffuse astrocytoma (IDH mutant and IDH wildtype).

### 2.2. MRI Structural Data

#### 2.2.1. Preoperative Tumor Volume

All the lesions were non-contrast enhancing LGG. Preoperative mean tumor volume (cm^3^) calculated on T2-weighted MRI was 16.54 ± 9.19 (range 5–40).

#### 2.2.2. Maximum Lesion Overlap

The maximum overlap of the lesion masks of patients with LH lesions mainly occurred in the left inferior frontal gyrus (pars opercularis).

**Figure 2:**
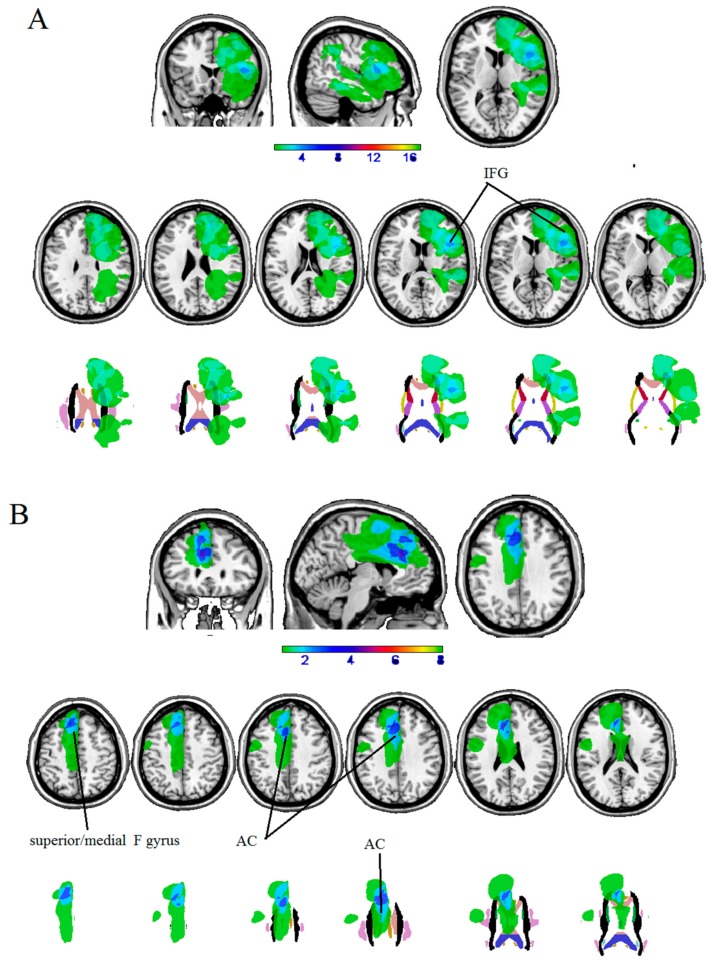
Overlaps of lesion masks of patients with (**A**) LH iLGG, and (**B**) RH ILGG at cortical (first row) and subcortical level (second row).

The maximum overlap of the lesion masks of patients with RH lesions mainly occurred in the right anterior cingulate and the right superior medial gyrus (see Table 5 and Figure 2).

**Figure 3:**
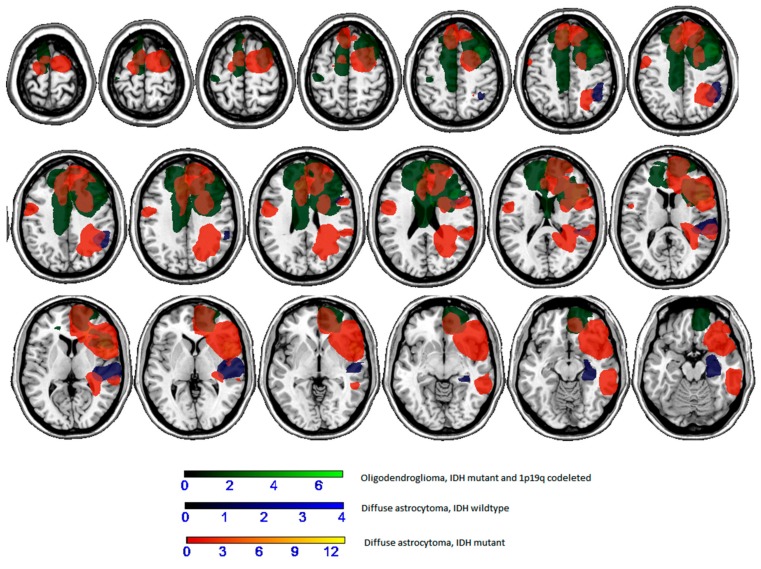
Overlaps of lesion masks according to molecular type.

As far as the molecular type is concerned, a preliminary analysis in which lesion masks were divided according to the molecular class showed that there was poor overlap between cases (see Figure 3). In general, patients with oligodendroglioma (IDH mutant and 1p/19q co-deleted) had lesions involving the cingulum (2 of 7) and the frontal cortex (2 of 7), among those with diffuse astrocytoma (IDH mutant) there was a lesion involving the parietal cortex or the superior temporal lobe (1 of 4), and those with diffuse astrocytoma (IDH wildtype) had lesions involving the insula and the frontal lobe (2 of 13).

## 3. Discussion

Incidental discovery of low-grade gliomas is rather uncommon, with an incidence of 0.04–0.2% in the total of asymptomatic subjects [4]. Other authors [24] reported a percentage of 0.47% of intracranial tumors in a group of 2536 healthy young adult males. The discovery of an incidental glioma may be a factor for a better prognosis for the patient, an early intervention and oncological treatment, and a higher extent of resection which could improve the patient’s life expectancy [16,20] by preventing a progression to a higher grade of malignancy [25].

We have studied a group of patients with iLGGs to evaluate their cognitive framework. From our monoinstitutional cohort of 332 patients with LGG we identified 11.52% as iLGGs. In our sample, iLGGs were found in a young (mean age 38.5 ± 1.06) population. We found a greater number of iLGGs in the left hemisphere, in contrast with what was found in the literature which indicates a higher involvement of the right hemisphere [20]. This inconsistency could be related to a bias since in our database there are more patients with a left hemispheric lesion.

In our iLGGs sample, iLGGs patients were cognitively preserved. There were also some borderline scores, which could be signs of initial cognitive worsening. Nonetheless, the areas involved in the iLGGs lesions are highly involved in cognitive functioning. We found that the maximum overlap of LH iLGGs lesion masks occurred in pars opercularis of the left inferior frontal gyrus (i.e., Broca’s area), and in the right anterior cingulate and the right superior medial gyrus for RH iLGGs patients. It follows that language and attention/inhibition related functions were at risk of possible decrements. These results indicate that at this time of tumor growth, cognition was still functional, but was slowly approaching the borderline performance. One reason could be tumor volumes: lesions were relatively circumscribed, ranging from 5 to 40 cm^3^. Another possibility is that plasticity, as a mechanism of preservation of cognitive functions, may have contributed through compensation to the preservation of cognition. Further studies are needed to directly target this issue by analyzing pre-surgery fMRI data.

The neuropsychological pattern could also be related to the iLGGs patients’ molecular class: a preliminary correlation analysis showed that, for visual–spatial intelligence and for visual–spatial short-term memory test, patients with glioma characterized by a mostly compact growth (such as the oligodendroglioma) had higher equivalent scores, corresponding to better performance with respect to those with diffuse astrocytoma. A preliminary analysis in which lesion masks were divided according to the molecular class showed that there was poor overlap between cases. This investigation deserves further studies on a larger sample. Indeed, lesion distribution could help the understanding between the observed differences in equivalent scores.

The fact that there are results scattered below the normal range performance is not frankly pathological; despite the presence of a brain lesion, patients do not detect any change in their cognitive functioning. Nonetheless these scattered altered performances are of relevance. While in symptomatic LGG different factors can contribute to cognitive dysfunction, like epilepsy, medication, or other focal neurologic sign [26], in our patients, neuropsychological deficits are obviously related to the tumor itself since none of our patient manifested symptoms, had seizures, or were in drug treatment. Therefore, any scattered decrement in neuropsychological performance indicates that the pathological tissue starts to compromise its functional role. Future studies will address the comparison of the iLGGs performance with clinically overt LGGs performance.

## 4. Materials and Methods

### 4.1. Participants

We retrospectively reviewed adult Caucasian patients who underwent surgery for infiltrative glioma between 2008 and 2019.

Inclusion criteria were: age ≥18 years; having a LGG; had an iLGG defined as gliomas found on imaging studies obtained for a reason unrelated to the underlying tumor, including headache (during the diagnostic workup of episodic primary headache, mainly migraine-like), trauma, otolaryngology disorders, or magnetic resonance imaging (MRI) studies [16] received a neuropsychological evaluation (only 24 out of 34 received neuropsychological evaluation).

In all cases, diagnostic brain MRI was performed before surgery, including a hydrogen magnetic resonance spectroscopy (H-MRS) examination

The present study was approved by the local ethics committee (protocol N. 0036567/P/GEN/EGAS, ID study 2540). Written informed consent was obtained for surgery. Considering that the study was retrospective, written consent to participate in the study was not applicable.

### 4.2. Neuropsychological Evaluation

Patients completed the neuropsychological testing conducted by a neuropsychologist prior to surgery on the same day as they performed fMRI. General questions about demographic characteristics like age, education level, employment status, comorbidities, clinical history and handedness were asked to all patients.

The main issue in clinical studies concerns the extent and site of anatomical lesions. In most patients with vascular etiology, cerebral lesions are not limited to a single lobe but encompass several brain structures. Neurosurgical patients, by contrast, usually suffer relatively circumscribed lesions within the left cerebral hemisphere. Therefore, several cognitive domains were considered: logical reasoning, psychomotor speed and attention processing, short-term memory and working memory, language, executive functioning, visual–constructive abilities and visual–spatial cognition. Not all participating subjects completed the same test battery, in relation to the site of the lesion. The neuropsychological battery for the group with left iLGGs included: Raven’s Matrices [27], objects and verbs naming [28,29], word and pseudoword repetition and reading, lexical decision, noun and verb comprehension, phonemic discriminations, auditory and visual lexical decisions [29], Palm and Pyramids [30], oral apraxia [31], ideomotor apraxia [32], Token Test [33], digit span forward and backward [34], Trail Making Test A/B [35], verbal fluency [36]. The neuropsychological battery for the group with right iLGG included: Raven’s Matrices [27], clock test [37], Corsi forward and backward [34], Rey–Osterrieth complex figure [38], Digit Symbol Substitution Test (Wechsler Adult Intelligence Scale-Revised) [39], letter cancellation, star cancellation, barrage, line bisection (Behavioral Inattention Test) [40], Trail Making Test A/B [35].

### 4.3. MRI Structural Data

Topographic and volumetric descriptions of the tumor were obtained by retrospectively analyzing structural imaging data routinely acquired during pre-surgery investigations. A 3-T Philips Achieva whole-body scanner was used to acquire structural data using a SENSE-Head-8 channel head coil. Volumes of interest (VOIs) of the patients’ lesions were drawn on their T1 MRI scans using MRIcron software (https://www.nitrc.org/projects/mricron). We then normalized the Region of Interests (ROIs) to the Montreal Neurological Institute (MNI) space using the “Clinical Toolbox” (https://www.nitrc.org/projects/clinicaltbx/) for SPM8 (https://www.fil.ion.ucl.ac.uk/spm/). The MRIcron procedure was used to overlap lesion masks (VOIs) (https://www.nitrc.org/projects/mricron). The output is a percentage overlay plot showing the percentage of overlapping lesions on a color scale.

### 4.4. Histological and Molecular Analysis

iLGGs patients were surgically treated using the same protocol used with symptomatic LGGs [6,16]. Histological and Molecular data were retrospectively analyzed (see [16]) according to the 2016 WHO classification.

### 4.5. Data Analysis

Patients with below the normal range performance were classified according to the published cut-off of each clinical test. We considered equivalent scores as an additional measure: patients’ scores were converted into equivalent scores using a 0–4 scale (in which 0 corresponds to a score below the 5% tolerance limits, 4 corresponds to a score equal to or better than the mean, and 1/2/3 correspond to intermediate scores between 0 and 4) according to the standardization of the tests.

Lastly, we performed a correlation analysis between patients’ equivalent scores (0–4, see above) and their molecular class (classified as 0/1/2). Analyses were performed by SPSS for Windows (version 12.0).

## 5. Conclusions

These data could be very relevant with respect to surgical strategy. It is known that at the present time there is no single protocol that defines how to intervene in the case of iLGGs. On one hand, there are numerous evidences supporting a positive impact of the maximum possible resection in the case of symptomatic LGG. However, the lack of understanding of history and the scarcity of data on iLGG, contributes to the dilemma of how they should be managed from the neurosurgical point of view [3]. In this perspective, the information about an iLGG patient’s neuropsychological status could become an element to consider in the decision-making strategy: a decreased or borderline performance could support the importance of an early intervention, performed before observing a progression of decrease in neuropsychological functions. Nevertheless, in our opinion, the oncological criteria should have priority in the decision-making process.

## Figures and Tables

**Table 1:** iLGGs patients’ demographic and clinical data.

H	P#	WHO_2016	Age	Ed	Site Surgery	H	V
LH	1	Diffuse astrocytoma, IDH mutant	42	13	F		10
LH	2	Oligodendroglioma, IDH mutant and 1p/19q co-del	40	13	F	100	18
LH	3	Diffuse astrocytoma, IDH wildtype	29	13	F		8
LH	4	Diffuse astrocytoma, IDH mutant	20	13	F	100	12
LH	5	Oligodendroglioma IDH mutant, 1p19q co-del	52	13	F	100	26
LH	6	Diffuse astrocytoma, IDH mutant	25	13	F	−100	15
LH	7	Diffuse astrocytoma, IDH wildtype	20	13	T	100	32
LH	8	Diffuse astrocytoma, IDH mutant	32	8	F	100	40
LH	9	Diffuse astrocytoma, IDH mutant	61	18	T	100	12
LH	10	Diffuse astrocytoma, IDH wildtype	46	11	T	50	12
LH	11	Diffuse astrocytoma, IDH mutant	40	16	F	100	34
LH	12	Diffuse astrocytoma, IDH wildtype	29	13	P	100	16
LH	13	Diffuse astrocytoma, IDH mutant	32	17	P	100	10
LH	14	Oligodendroglioma, IDH mutant and 1p/19q co-del	48	13	F	100	12
LH	15	Diffuse astrocytoma, IDH mutant	28	11	T	100	10
LH	16	Oligodendroglioma IDH mutant, 1p19q co-del	52	13	F	83	5
RH	1	Diffuse astrocytoma, IDH mutant	30	18	F	100	18
RH	2	Oligodendroglioma, IDH mutant and 1p/19q co-del	35	18	F	100	22
RH	3	Diffuse astrocytoma, IDH mutant	26	17	F		14
RH	4	Diffuse astrocytoma, IDH mutant	30	18	F	100	18
RH	5	Diffuse astrocytoma, IDH mutant	71	8	F	100	8
RH	6	Diffuse astrocytoma, IDH mutant	45	8	F	100	27
RH	7	Oligodendroglioma, IDH mutant and 1p/19q co-del	49	13	F	100	8
RH	8	Oligodendroglioma, IDH mutant and 1p/19q co-del	25	13	F	100	10

H = hemisphere; P# = case number; Ed = Education; H = handedness; V = Preoperative tumor volume (cm^3^) T2 weighted MRI.

**Table 2:** Reasons for initial MRI by which the incidental low-grade gliomas (iLGGs) were incidentally discovered.

Reason to Perform MRI	Number of Cases
Headache *	4
Otolaryngology disorders ^	3
Mental confusion	1
Hearing disturbance	2
Sensitive disorders ^	6
Dizziness	2
Other	2

* Headache includes migraine, tension-type headache. Patients who had a headache with any sign of increased intracranial pressure were excluded. ^ None of the iLGG patients experienced epilepsy and their pre-surgery EEG was normal.

**Table 3:** Left hemisphere (LH) and right hemisphere (RH) iLGG patients’ neuropsychological profile. Patients performed different series of tasks according to the hemisphere involved by the lesion. Performance below the normal range is highlighted dark grey. Equivalent scores equal to 1, indicating borderline performance are highlighted in light grey. Patients’ scores were converted into equivalent scores using a 0–4 scale, in which 0 corresponds to a score below the 5% tolerance limits, 4 corresponds to a score equal to or better than the mean, and 1/2/3 correspond to intermediate scores between 0 and 4, according to the standardization of the tests.

Neuropsychological Evaluation																				
		Intelligence	Memory		Praxis		Language			Executive Functions				Memory		Visuo-Spatial				Psychomotor Speed
H	P#	RAVEN	V_STM	WM	O_prax	IMA	V_comp	N_nam	V_nam	Ph_Flu	TMT A	TMT B	TMTB-A	S_STM	S_WM	C_pra	Clock	Bit_St	Line_bis	Dig_Sym
LH	1	35	5,6	2,6	19,75	Na	32,5	29	23	26	32	135	117	Na	Na	Na	Na	Na	Na	Na
LH	2	33	5,6	3,6	19,75	72	33,5	30	28	31	23	76	53	Na	Na	Na	Na	Na	Na	Na
LH	3	32	5,5	4,5	19,75	Na	33,5	30	28	62	Na	Na	Na	Na	Na	Na	Na	Na	Na	Na
LH	4	33	8,4	3,4	19,75	72	33,5	28	28	45	Na	Na	Na	Na	Na	Na	Na	Na	Na	Na
LH	5	Na	3,6	3,6	19,75	71	29,8	29	24	40	Na	Na	Na	Na	Na	Na	Na	Na	Na	Na
LH	6	31	5,4	4,4	19,75	72	33,5	29	28	20	Na	Na	Na	Na	Na	Na	Na	Na	Na	Na
LH	7	26	4,4	2,4	Na	72	33,5	30	28	49	Na	Na	Na	Na	Na	Na	Na	Na	Na	Na
LH	8	27	4,7	3,8	19,75	72	34,8	29	27	20	33	93	65	Na	Na	Na	Na	Na	Na	Na
LH	9	34	5,8	4,7	20	72	31,5	Na	28	49	Na	Na	Na	Na	Na	Na	Na	Na	Na	Na
LH	10	34	Na	Na	19,75	72	33,5	30	28	34	Na	Na	Na	Na	Na	Na	Na	Na	Na	Na
LH	11	31	7,5	4,4	19,75	72	32,8	29	28	29	Na	Na	Na	Na	Na	Na	Na	Na	Na	Na
LH	12	Na	Na	Na	19,75	72	33,5	30	27	40	Na	Na	Na	Na	Na	Na	Na	Na	Na	Na
LH	13	30	4,4	3,3	19,75	Na	32,8	28	27	34	46	137	180	Na	Na	Na	Na	Na	Na	Na
LH	14	33	4,8	3,7	19,75	Na	34	29	27	29	25	92	67	Na	Na	Na	Na	Na	Na	Na
LH	15	Na	5,4	Na	19,75	Na	Na	23	Na	Na	Na	Na	Na	Na	Na	Na	Na	Na	Na	Na
LH	16	Na	7	Na	19,75	Na	Na	28	Na	Na	Na	Na	Na	Na	Na	Na	Na	Na	Na	Na
RH	1	28	Na	Na	Na	Na	Na	Na	Na	Na	37	125	88	Na	Na	12	10	53	9	Na
RH	2	32	Na	Na	Na	Na	Na	Na	Na	Na	51	113	62	6,4	3,5	12	10	52	Na	62
RH	3	32	Na	Na	Na	Na	Na	Na	Na	Na	44	141	96	5,3	5,4	12	10	Na	9	47
RH	4	30	Na	Na	Na	Na	Na	Na	Na	Na	61	156	95	5,3	3,4	12	10	54	9	46
RH	5	30	Na	Na	Na	Na	Na	Na	Na	Na	27	26	-1	4,4	4,3	13	10	52	9	50
RH	6	28	Na	Na	Na	Na	Na	Na	Na	Na	31	70	39	4,9	3,7	13	10	54	9	48
RH	7	36	Na	Na	Na	Na	Na	Na	Na	Na	27	53	26	5,7	4,8	13	10	Na	9	57
RH	8	28	Na	Na	Na	Na	Na	Na	Na	Na	Na	Na	Na	Na	Na	Na	8,5	54	Na	Na

Hemi = hemisphere; P#= case number; LH = left hemisphere; RH = right hemisphere; Na = not administered; V_STM = verbal short-term memory; WM = working memory; O_prax = oral praxis; IMA = ideomotor apraxia; V_comp = verbal comprehension (Token Test); N_nam = noun naming; V_nam = verb naming; Ph_Flu = phonological fluency; TMT = Trail Making Test; S_STM = spatial short-term memory; S_WM = spatial working memory; C_pra = constructional apraxia; Clock = clock test; Bit_St = Behavioral Inattention test_star cancellation; Line_bis = line bisection; Dig_Sym = Digit Symbol Substitution Test.

**Table 4:** LH and RH iLGG patients’ additional neuropsychological test, according to the lesion site.

H	P#	Au_N_Comp	Au_V_comp	P&P	W_Read	Pw_Read	W_Rep	Pw_Rep	W_Wri	Pw_Wri	Phon_Disc	Au_lex-dec	Vis_lex-dec	Rey_C	Rey_R	BIT Let
LH	1	40	20	Na	45	45	45	20	15	25	60	88	99	Na	Na	Na
LH	2	Na	Na	Na	45	45	45	35	25	25	60	80	80	Na	Na	Na
LH	3	40	20	50	45	45	45	35	25	25	60	80	80	Na	Na	Na
LH	4	40	20	49	45	45	45	35	25	25	60	79	77	Na	Na	Na
LH	9	40	20	49	Na	45	45	35	25	25	60	80	80	Na	Na	Na
LH	11	40	20	48	45	45	45	35	25	25	60	80	80	Na	Na	Na
LH	13	40	20	48	45	45	45	35	25	25	60	80	80	Na	Na	Na
RH	3	Na	Na	Na	Na	Na	Na	Na	Na	Na	Na	Na	Na	33	24	Na
RH	4	Na	Na	Na	Na	Na	Na	Na	Na	Na	Na	Na	Na	Na	Na	35
RH	5	Na	Na	Na	Na	Na	Na	Na	Na	Na	Na	Na	Na	Na	Na	36
RH	6	Na	Na	Na	Na	Na	Na	Na	Na	Na	Na	Na	Na	35	5,3	36

Hemi = hemisphere; P#= case number; LH = left hemisphere; RH = right hemisphere; Na = not administered; Au_N_comp = Auditory noun comprehension; Au_V_comp = Auditory verb comprehension, P&P = Palm and pyramids test; W_Read = word reading; Pw_Read = pseudoword reading; W_Rep = word repetition; Pw_Rep = pseudoword repetition; W_Wri = word writing, Pw_Wri = pseudoword writing; Phon_Disc = phonologic discrimination; Au_lex-dec = auditory lexical decision; Vis_lex-dec = visual lexical decision; Rey_C = Rey–Osterrieth complex figure test (Copy immediate); Rey_R = Rey–Osterrieth complex figure test (Remember); BIT Let = Behavioral inattention test.

**Table 5:** Montreal Neurological Institute (MNI) coordinates of the maximum lesion volumes of interest (VOIs) overlap.

Area	Hemisphere	MNI			% Overlap
		x	y	z	
Inferior Frontal Gyrus (opercular)	LH	−46	15	13	23.52%
Anterior cingulate	RH	9	35	27	50%
Superior medial gyrus	EH	9	36	50	37.5%
